# Response to SARS-CoV-2 vaccination in multiple sclerosis patients on disease modifying therapies

**DOI:** 10.1007/s00415-022-11053-7

**Published:** 2022-03-14

**Authors:** M. Allman, E. Tallantyre, N. P. Robertson

**Affiliations:** 1 Department of Neurology, University Hospital of Wales, Heath Park, Cardiff, CF14 4XN UK; 2 Division of Psychological Medicine and Clinical Neuroscience, Department of Neurology, Cardiff University, University Hospital of Wales, Heath Park, Cardiff, CF14 4XN UK

## Introduction

The COVID-19 pandemic has caused significant mortality and morbidity throughout the world, with vaccination against the SARS-CoV-2 virus now widely considered the most effective strategy to minimise clinical impact. However, people with multiple sclerosis (pwMS), treated with disease modifying therapies (DMTs), which target and supress varying components of the immune response, are considered more vulnerable to severe disease. Furthermore, recovery and immunity from COVID-19 relies on an effective immune response to specific components of the SARS-CoV-2 virus and it is unclear whether pwMS on DMTs are able to consistently develop adequate, durable protection. This month’s journal club describes four papers that aim to answer questions surrounding this issue.

## Brill L, Rechtman A, Zveik O, et al. Humoral and T-cell response to SARS-CoV-2 vaccination in patients with multiple sclerosis treated with ocrelizumab

This was a single centre prospective cohort study which included 72 patients with MS, 49 of whom were treated with ocrelizumab, 23 who were untreated for at least 6 months, and 40 healthy controls. Study participants received two doses of BNT162b2 vaccine (Pfizer/BioNTech). Antibody and T-cell responses were assessed at baseline and 2–4 weeks after second dose.

Mean antibody rates were significantly lower in patients treated with ocrelizumab (Mean 26.2 and 376.5 AU/mL) compared with healthy controls (Mean 283 and 12,712 AU/mL) and untreated patients (mean 288.3 and 10,877 AU/mL). 8 patients treated with ocrelizumab did not reach a positive serology threshold in any of their samples. There was a positive association between SARS-CoV-2 IgG levels and time from last ocrelizumab dose. Patients who were vaccinated 5 or more months from their last ocrelizumab dose had a significantly increased likelihood of a positive serological response. There was positive SARS-CoV-2 specific T-cell responses in 26 out of 29 patients (89.7%) on ocrelizumab and all of the vaccinated controls. There was no significant difference in the number of responding T-cells between vaccinated MS patients on ocrelizumab and healthy controls. There was no correlation between antibody response and T-cell response.

**Comment:** This study helps to inform decisions regarding vaccination in MS patients treated with ocrelizumab. It has identified the significance of timing of last ocrelizumab dose when deciding vaccination schedules and also provided some encouraging data on T-cell response in these patients. The study does not define clearly the extent to which T-cell responses are responsible for immunity against COVID-19, although robust T-cell responses have been associated with mild or asymptomatic COVID-19 infection and viral-specific T-cell response can help to kill viral-infected cells.

Possible weaknesses of the study include a small sample size, and it is, therefore, difficult to confidently extrapolate these results to the wider population, the absent of demographic variables to determine representiveness, short duration of follow-up and absence of matching between cases and controls.

*JAMA Neurol. 2021;78(12):1510–1514*

## Tortorella C, Aiello A, Gasperini C, et al. Humoral- and T-cell-specific immune responses to SARS-CoV-2 mRNA vaccination in patients with MS using different disease-modifying therapies

This is a prospective cohort study of 186 vaccinated individuals consisting of 108 patients with MS and 78 healthcare workers (HCWs). MS patients were being treated with various disease-modifying treatments (DMTs) including interferon-β (35), fingolimod (20), cladribine (20) and ocrelizumab (25). The aim of the study was to evaluate the neutralizing anti-RBD antibodies and spike-specific T-cell response after a full course of SARS-CoV-2 vaccinations.

Ocrelizumab, fingolimod and cladribine treated patients demonstrated lower response rates and antibody titres compared to HCWs. A detectable anti-RBD response was seen in 40% treated with ocrelizumab, 85.7% on fingolimod, 96.4% on IFN-β, and 100% on cladribine. There was no significant difference in the median titre of patient on IFN-β when compared with HCWs (*p* = 0.359). Length of treatment with ocrelizumab was significantly associated with reduced anti-RBD titres, but no correlation between time elapsed since the last treatment cycle and antibody titre. Neutralising antibodies were present in the serum of all the HCWs. However, only 4/24(16.6%) fingolimod-treated MS patients showed neutralising activity, and these results were significantly correlated with antibody titres (*ρ* = 0.591, *p* = 0.0024). Patients on other DMTs were not tested as their antibody titre was too low.

The T-cell specific response was significantly lower in MS patients compared with HCWs (*p* < 0.0001). All HCWs showed a spike-specific T-cell response, compared with 62% of MS patients. There were statistically significant differences in response between DMTs, with 92% of patients on ocrelizumab, 70% on cladribine, 89.3% in the IFN-β group and 14.3% on fingolimod (*p* < 0.0001).

**Comment:** This study has provided useful data to inform vaccine management in pwMS and highlights the need to continue to promote vaccination in this patient group. As in the previous study, the authors recommend considering alterations to the vaccination schedule to account for the last dose of ocrelizumab, to improve immune response.

This study had clear inclusion criteria and demographic variables available for comparison between patients and controls. They also adjusted for age, sex, BMI, and disease duration. This makes it more likely that researchers will be able to capture true differences between the immune response of patients on different DMTs. However, the sample size is once again small, especially when considering the numbers of patients on individual DMTs. Additional useful data is also provided on neutralising antibodies, which has practical implications for evaluating patients’ immunity to COVID-19. The study did not assess pre-vaccination antibody levels, as enrolment was post-vaccination, and this could have a significant impact on post-vaccination antibody titres. The authors also emphasise that a T-cell response alone does not necessarily provide sufficient defence against SARS-CoV-2 infection and further work is still required to establish this.

*Neurology. 2022 Feb 1;98(5):e541–e554*

## Sormani MP, Inglese M, Schiavetti I, et al. Effect of SARS-CoV-2 mRNA vaccination in MS patients treated with disease modifying therapies

This prospective multi-centre cohort study was conducted across 35 Italian MS centres enrolling 780 patients with MS; 87 untreated, 154 treated with ocrelizumab, 25 on rituximab, 85 on fingolimod, 25 on cladribine and 404 on other DMTs. All participants had blood taken prior to vaccination and at 4 weeks after second dose. The primary aim of the study was to quantify the level of SARS-CoV-2 RBD antibodies elicited by vaccination according to DMT. The study also assessed relationship between antibody levels post-vaccination and time since last infusion of an anti-CD20 agent. All patients received two vaccine doses: 594 with BNT 162b2 and 186 with mRNA-1273 vaccine.

780 participants underwent post-vaccination blood sampling; 73(9.4%) were positive for RBD or nucleocapsid antibodies before vaccination (evidence of prior infection), 677/780(86.8%) were positive for RBD after vaccination and 68/73 participants who were already positive showed a fourfold increase in RBD antibodies. Antibody responses varied significantly between DMTs. All untreated patients or those on cladribine, glatiramer acetate, alemtuzumab, dimethyl-fumarate and teriflunomide mounted a full response, and only one patient (1.3%) on interferon did not. However, absent antibody response occurred in 7.1% (*N* = 6) on fingolimod, 56.5% (*N* = 87) on ocrelizumab and 36.0% (*N* = 9) on rituximab.

Factors significantly associated associated with post-vaccination antibody titres were; pre-vaccination antibodies, type of DMT, and type of vaccine. Vaccination with mRNA-1273 resulted in a systematically 3·25-fold higher antibody level (95%CI = 2·46–4·27) than with BNT162b2 vaccine (*p* < 0·001). Being on ocrelizumab (201-fold decrease), fingolimod (26-fold decrease) and rituximab(20-fold decrease) were all significantly associated with reduced RBD antibodies (*p* < 0.001). A longer interval between last dose of ocrelizumab and rituximab and vaccination was also associated with a higher antibody titre, and suggested that an interval of 143 days (95% CI 84–258) is required to mount an antibody response to the vaccine.

**Comment:** This study had a large sample size which ensured results had sufficient power to detect true differences between DMTs and effectively controlled for multiple variables in the analysis. Antibody levels were checked pre-vaccination, and therefore, it was possible to account for pre-existing immunity. These preliminary results were from the initial short follow-up, at 4 weeks; however, further follow-up is planned for 6 and 18 months which may be important as some evidence suggest that antibody titres peak at 8 weeks.

This study had no healthy control group for comparison and excluded patients if they were on more than one immunomodulating treatment and only assessed humoral response, rather than T-cell mediated response.

Findings from this well-conducted study confirms previous data on the partial lack of antibody response in pwMS on anti-CD20 medications. It also suggests that type of SARS-CoV-2 vaccine may be important, as the mRNA-1273 (Moderna) elicited a significantly higher antibody response than BNT162b2 vaccine (Pfizer/BioNTech). It confirms benefits of a longer time interval from last dose of anti-CD20 medication to vaccination, and the authors make a recommendation of at least 143 days. Finally, the study noted a fourfold increase in antibodies in those who already had antibodies present which may indicate the potential for improved humoral response following booster doses of the vaccine.

*EBioMedicine. 2021 Oct;72:103581.* https://doi.org/10.1016/j.ebiom.2021.103581

## Pitzalis M, Idda ML, Lodde V, et al. Effect of different disease-modifying therapies on humoral response to BNT162b2 vaccine in sardinian multiple sclerosis patients

This is a rospective cohort study across MS centres in Sardinia, Italy, enrolling 912 MS patients and 63 healthy controls. All patients received two doses of BNT162b2 vaccine. Blood samples were collected for analysis 30 days after second vaccination and 613 patients also had blood samples taken prior to their first dose. 205 (22.5%) MS patients were untreated, 161 dimethyl fumarate, 135 interferon, 96 glatiramer acetate, 75 fingolimod, 75 natalizumab, 56 teriflunomide, 42 ocrelizumab, 17 alemtuzumab, 13 rituximab, 6 cladribine and 2 methotrexate. Analysis was not carried out on DMTs with less than 10 patients. A negative binomial generalised linear mixed-effects model was used to explore differences between antibody response in treated and untreated MS patients, and the control group.

The study found no significant different between antibody response in untreated MS patients and healthy controls, suggesting MS disease alone does not play a significant role in the vaccine response. There was no significant differences in Anti-S antibodies between untreated patients and patients on dimethyl fumarate, interferons, alemtuzumab, or glatiramer acetate, all of whom had high levels of antibodies post-vaccination. However, patients treated with teriflunomide, azathioprine, natalizumab, ocrelizumab and rituximab had a significantly lower level of Anti-S antibodies post-vaccination.

Time since last dose of anti-CD20 medication also had a significant effect on antibody production and the authors concluded that if vaccination was > 6 months after last dose then there was a siginificant upregulation in antibody production (*p* = 0.012). Patients with evidence of a previous infection,had significantly higher antibody levels than those without (14,000 v 1040U/mL *p* = 2.27E–18). Additional factors contributing to antibody production were also noted: males, smokers and older patients all having significantly reduced anti-S antibody titres.

**Comment:** This study adds to evidence demonstrating a reduction in antibody production in pwMS on anti-CD20 medications and fingolimod, but high levels of antibody production following BNT162b2 vaccination in patients on other DMTs. This study provided classification of disease course in study participants (82.7% relapsing–remitting MS, 1.8% primary-progressive MS and 15.5% secondary-progressive MS) and also analysed for differences in antibody production based on age, gender, smoking and disability score which may inform personalised management decisions. The significant increase in humoral response in patients with previous natural infection is also an important observation and once again suggests a value for booster vaccinations.

*Front Immunol. 2021 Dec 9;12:781843.* https://doi.org/10.3389/fimmu.2021.781843

